# Relative importance of triglyceride glucose index combined with body mass index in predicting recovery from prediabetic state to normal fasting glucose: a cohort analysis based on a Chinese physical examination population

**DOI:** 10.1186/s12944-024-02060-w

**Published:** 2024-03-08

**Authors:** Hongyi Yang, Maobin Kuang, Jiajun Qiu, Shiming He, Changhui Yu, Guotai Sheng, Yang Zou

**Affiliations:** 1 Department of Ultrasound, the Second Affiliated Hospital of Nanchang University, Nanchang, Jiangxi Province China; 2 Jiangxi Medical College, Nanchang University, Nanchang, Jiangxi Province China; 3 Jiangxi Cardiovascular Research Institute, Jiangxi Provincial People’s Hospital, The First Affiliated Hospital of Nanchang Medical College, Nanchang, Jiangxi Province China; 4 Jiangxi Provincial Geriatric Hospital, Jiangxi Provincial People’s Hospital, The First Affiliated Hospital of Nanchang Medical College, Nanchang, Jiangxi Province China

**Keywords:** Triglyceride-Glucose index, Body mass index, Prediabetes regression, Prediabetes recovering, Recovery from prediabetes

## Abstract

**Background:**

Prediabetes is a high-risk state for diabetes, and numerous studies have shown that the body mass index (BMI) and triglyceride-glucose (TyG) index play significant roles in risk prediction for blood glucose metabolism. This study aims to evaluate the relative importance of BMI combination with TyG index (TyG-BMI) in predicting the recovery from prediabetic status to normal blood glucose levels.

**Methods:**

A total of 25,397 prediabetic subjects recruited from 32 regions across China. Normal fasting glucose (NFG), prediabetes, and diabetes were defined referring to the American Diabetes Association (ADA) criteria. After normalizing the independent variables, the impact of TyG-BMI on the recovery or progression of prediabetes was analyzed through the Cox regression models. Receiver Operating Characteristic (ROC) curve analysis was utilized to visualize and compare the predictive value of TyG-BMI and its constituent components in prediabetes recovery/progression.

**Results:**

During the average observation period of 2.96 years, 10,305 individuals (40.58%) remained in the prediabetic state, 11,278 individuals (44.41%) recovered to NFG, and 3,814 individuals (15.02%) progressed to diabetes. The results of multivariate Cox regression analysis demonstrated that TyG-BMI was negatively associated with recovery from prediabetes to NFG and positively associated with progression from prediabetes to diabetes. Further ROC analysis revealed that TyG-BMI had higher impact and predictive value in predicting prediabetes recovering to NFG or progressing to diabetes in comparison to the TyG index and BMI. Specifically, the TyG-BMI threshold for predicting prediabetes recovery was 214.68, while the threshold for predicting prediabetes progression was 220.27. Additionally, there were significant differences in the relationship of TyG-BMI with prediabetes recovering to NFG or progressing to diabetes within age subgroups. In summary, TyG-BMI is more suitable for assessing prediabetes recovery or progression in younger populations (< 45 years old).

**Conclusions:**

This study, for the first time, has revealed the significant impact and predictive value of the TyG index in combination with BMI on the recovery from prediabetic status to normal blood glucose levels. From the perspective of prediabetes intervention, maintaining TyG-BMI within the threshold of 214.68 holds crucial significance.

**Supplementary Information:**

The online version contains supplementary material available at 10.1186/s12944-024-02060-w.

## Background

Prediabetes, defined by blood glucose levels higher than normal but below the threshold for diabetes, is also referred to as non-diabetic hyperglycemia or moderate hyperglycemia [1–3]. The term prediabetes was initially used to designate individuals with a high likelihood of developing diabetes in the future [4]. However, with advancements in research, prediabetes is now recognized as a marker indicating heightened risk for future kidney disease, microvascular complications, small fiber neuropathy, tumors, vascular diseases [1–3]. It is also closely linked to accelerated bone loss [5], rapid cognitive aging [6], and even an increased risk of mortality [5]. Fortunately, most cases of hyperglycemia in prediabetes are recoverable. Several randomized controlled trials investigating drug interventions or lifestyle modifications have reported research outcomes demonstrating the recovery of prediabetes to normal blood glucose levels [7–17]. In summary, recovery from prediabetes is beneficial, as the significant return to normal blood glucose levels reduces the risk of diabetes and various long-term complications in study participants. Given that there are over 400 million people worldwide in a prediabetic state [18], it will be of great benefit to reverse the situation and the consequent benefits if the changeable factors that affect the return to normal blood glucose in pre-diabetes can be identified early.

Over the past few decades, disease progression-related research, including prediabetes, has received widespread attention worldwide [1–3]. Relatively less research, however, has focused on the process of recovery from prediabetes to normal blood glucose [19]. From the perspective of disease development, insulin resistance (IR) and β-cell dysfunction are key factors in the progression from normal blood glucose to prediabetes [1, 20, 21]. From a treatment standpoint, improving IR and β-cell function are key elements in facilitating the transition from prediabetes to normal blood glucose [22]. This bidirectional evidence further underscores the importance of effectively assessing IR status for the prevention and management of prediabetes. Currently, the hyperinsulinemic-euglycemic clamp technique is upheld as the gold standard for measuring IR in the field [23]. However, due to its technical complexity and invasiveness, this technique is greatly limited in clinical practice and disease screening [24]. TyG-BMI is an alternative for assessing IR, developed in recent years. It builds upon the IR surrogate index TyG index and further considers the potential impact of obesity [25]. According to the report of Ko et al., TyG-BMI has significantly higher value in assessing IR compared to traditional lipid measurement parameters, lipid ratios, adipokine, and the TyG index. Subsequently, in multiple longitudinal studies, researchers have found that TyG-BMI also has good predictive value in assessing prediabetes and diabetes progression [26–30]. However, it remains unclear whether TyG-BMI plays a significant role and to what extent it influences the process of recovery from prediabetes to normal blood glucose.

## Methods

### Data source and study population

This retrospective analysis of a longitudinal study employed health examination data from the Rich Healthcare Group in China on a national scale. The dataset, collected and compiled by Chen et al., includes adult health examination participants recruited by the Rich Healthcare Group (2010–2016) in 32 regions across 11 cities in China, and these subjects underwent health examinations at least twice during this period (*n* = 685,277). In the initial data analysis, Chen et al. investigated the association between BMI and the risk of future diabetes [31]. They excluded participants who had the following conditions: (i) participants with missing data at baseline, including sex, fasting plasma glucose (FPG), weight and height (*n* = 135,317); (ii) participants who had a confirmed diabetes diagnosis at the time of the first evaluation (*n* = 7,112); (iii) subjects with extreme BMI values (*n* = 152); (iv) participants with an interval of less than 2 years between two consecutive visits (*n* = 324,233); and (v) participants with unknown diabetes status during longitudinal observation (*n* = 6,630). In the end, Chen et al. evaluated 211,833 participants in their data analysis related to the association between BMI and diabetes. The dataset used for their research analysis has also been publicly shared on the DRYAD database by them [32]. According to the DRYAD database usage terms, the researchers can engage in secondary analysis based on the original data provided by Chen et al. after properly acknowledging the data source [32].

**Fig. 1:**
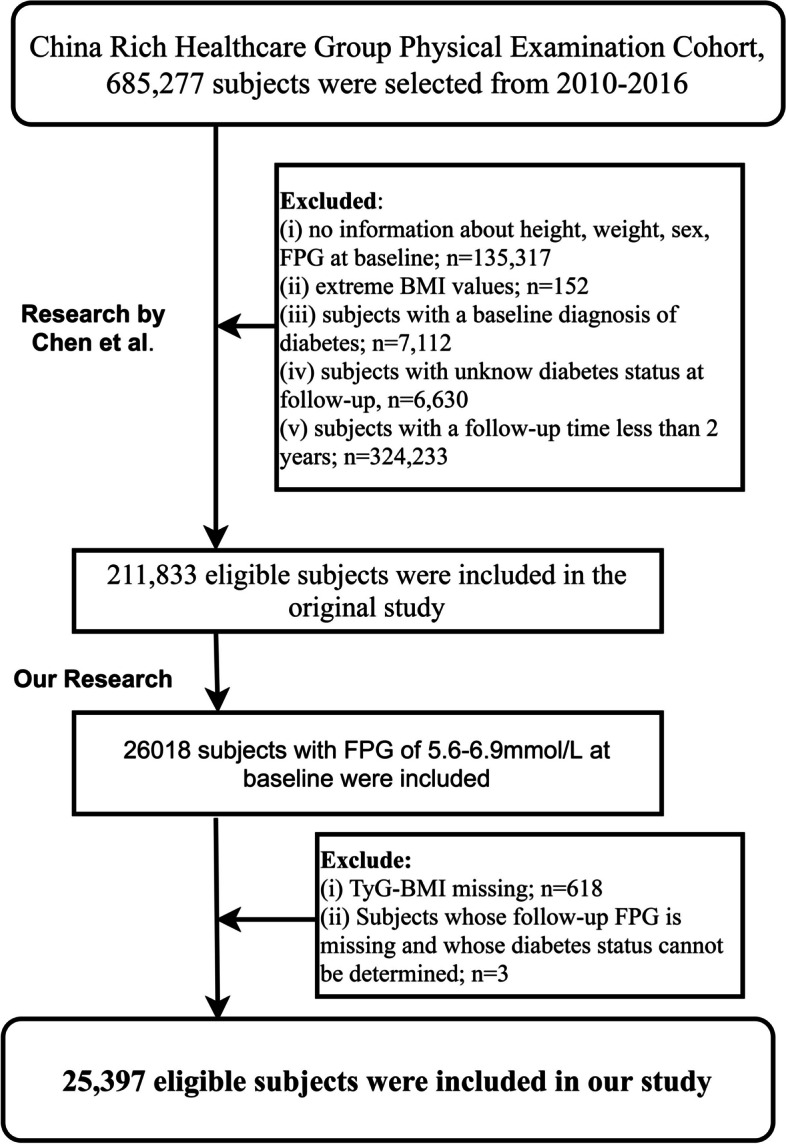
Flow chart of study participants

The research hypothesis of the current study: Does TyG-BMI play a role in the recovery of prediabetic status to normal blood glucose, and if so, to what extent? Based on the dataset analyzed by Chen et al. [32], this study further conducted the inclusion of the study population following the criteria outlined in Fig. 1, as detailed below: (i) Non-prediabetic subjects were excluded according to ADA's pre-diabetic diagnostic criteria [33] (ii) Participants with missing values for independent variables. (iii) Participants who lacked follow-up FPG data or had an unclear diabetes status. This research is a secondary analysis, a new research protocol has been submitted and approved by the Jiangxi Provincial People's Hospital Ethics Committee (No. 2021–067). Additionally, considering that the dataset has been anonymized, the ethics Committee of the author's institution waived the signing of the informed consent form of the participants. The entire secondary analysis process strictly adhered to the STROBE reporting guidelines, as detailed in Supplementary Table 1.

### Baseline data measurement and collection

As previously described in the earlier study [31], participants were required to complete a standardized questionnaire survey during each health screening, documenting their living habits (drinking/smoking status), the presence of chronic diseases (such as diabetes), and family history (family history of diabetes). Anthropometric measurements, including height, weight, and blood pressure (BP), were conducted by trained healthcare professionals in a standard environment. Among them, BP measurements were taken using a mercury sphygmomanometer, a trusted device for accurately measuring blood pressure. Participants were instructed to wear light clothing and take off their shoes for precise height and weight measurements, which were recorded to 1 decimal place. Biochemical measurements required participants to collect venous blood samples after at least 10 h on an empty stomach, which were subsequently analyzed in a standard laboratory using automated analytical instruments. The current dataset includes the following biochemical parameters: aspartate aminotransferase (AST), high-density lipoprotein cholesterol (HDL-C), alanine aminotransferase (ALT), triglycerides (TG), creatinine (Cr), total cholesterol (TC), blood urea nitrogen (BUN), low-density lipoprotein cholesterol (LDL-C) and FPG, in which FPG was measured by oxidase method.

### Calculation [25]

$${\mathrm {BMI = Weight(kg)/Height{(m)}^{2}}}$$

$${\mathrm {TyG-BMI=BMI* \ Ln\ [FPG \times\ TG/2]}}$$

### Follow-up and outcomes

The study population included individuals who were initially diagnosed with prediabetes at baseline. Any changes in blood glucose status that occurred during the follow-up period constituted the outcomes of interest, including the progressing to diabetes or recovery of prediabetes to NFG. The determination of NFG, prediabetes, and diabetes in the current analysis was based on the ADA’s diagnostic criteria, which relies on FPG levels [33]. Specific as follows: (i) NFG: During the observation period, FPG returned to below 5.6 mmol/L. (ii) Prediabetes: FPG levels ranging from 5.6 to 6.9 mmol/L. (iii) Diabetes: FPG levels exceeding 7.0 mmol/L during the observation period or self-reported diabetes diagnosis by other healthcare professionals.

### Statistical analysis

The study's analyses utilized R version 3.4.1 and Empower(R) version 2.0 software. A two-tailed *P*-value < 0.05 indicated statistical significance. For the description of baseline variables were grouped and summarized according to study outcomes. The type of baseline variable and type of distribution (Judging by histogram) were first assessed, and then in subsequent tables were reported as mean [standard deviation (SD)]/median (interquartile spacing)/frequency (%).

For the multi-classified study endpoints in the current study, the researchers used one-versus-rest to split the data to a binary dataset for each class [34, 35], and investigated the effect of TyG-BMI on the recovery/progression of prediabetes separately. As the main analytical method, stepwise-adjusted multivariate Cox regression models were used for the assessment of associations in the current study [36], and the hazard ratio (HR) per SD increase and its confidence interval (CI) were recorded. Furthermore, before establishing the models, the variance inflation factor was calculated for covariates to assess collinearity among covariates [37]. Three progressively adjusted multivariate regression models were constructed for the association analysis in the current study. Model I adjusted height, age and sex; Model II further considered the potential influence of lifestyle factors and BP; Model III made additional adjustments to FPG, TG, Cr, BUN and ALT based on model II. Based on Model III, the researchers also employed restricted cubic splines (RCS) in four knots nested within Cox regression to fit and visualize the relationship of TyG-BMI with the progression or recovery of prediabetes.

To validate the stability of the impact of TyG-BMI on the recovery/progression of prediabetes, the researchers conducted a series of sensitivity analyses: (i) TyG-BMI was categorized into quartiles and examined the trend of association between TyG-BMI and prediabetes recovery/progression based on the median of the categorical variable. (ii) According to the prediabetes and diabetes diagnostic criteria published by the WHO [38], the study subjects were re-included and excluded, and performed the same steps of association analysis. (iii) The occurrence of diabetes events as the study outcome precludes us from observing the potential recovering to NFG in this subset of the population. Therefore, the competing risk model was applied with diabetes events as the competing endpoint to assess the association of TyG-BMI and its components with prediabetes recovery or reversal. (iv) Considering the direct and indirect potential impact of a family history of diabetes on blood glucose [39, 40], the researchers conducted the same analysis in people without a family history of diabetes. (v) To account for potential unmeasured confounding, the researchers calculated the E-value [41]. (vi) 3 and 5 knots of RCS were used to test the linearity of the relationship of TyG-BMI with prediabetes recovery.

After determining the relationship of TyG-BMI with prediabetes recovery/progression, the researchers aimed to further assess the predictive power of TyG-BMI in prediabetes recovery/progression. To address this issue, the researchers conducted ROC analysis, calculating metrics such as the area under the curve (AUC), optimal threshold, sensitivity, and specificity, and compared the predictive value of TyG-BMI and its component parts for prediabetes recovery/progression using the DeLong test [42].

The researchers also conducted several subgroup analyses within several common phenotypic subgroups known to have a significant impact on blood glucose levels, including sex, age, and BMI. Age stratification followed WHO classification standards [43], while BMI stratification followed the guidelines suggested by the Chinese Obesity Working Group [44]. Likelihood ratio tests were used to assess interactions between TyG-BMI and the stratification variables.

## Results

### Follow-up results and study population characteristics

This study included a total of 25,397 participants initially diagnosed with prediabetes, with an average age of 49 years and males comprising 66.28% of the cohort. Over an average observation period of 2.96 years, among the 25,397 participants, 10,305 individuals (40.58%) remained in the prediabetic state, 11,278 individuals (44.41%) experienced a return to normal blood glucose levels, and 3,814 individuals (15.02%) progressed to diabetes.

**Fig. 2:**
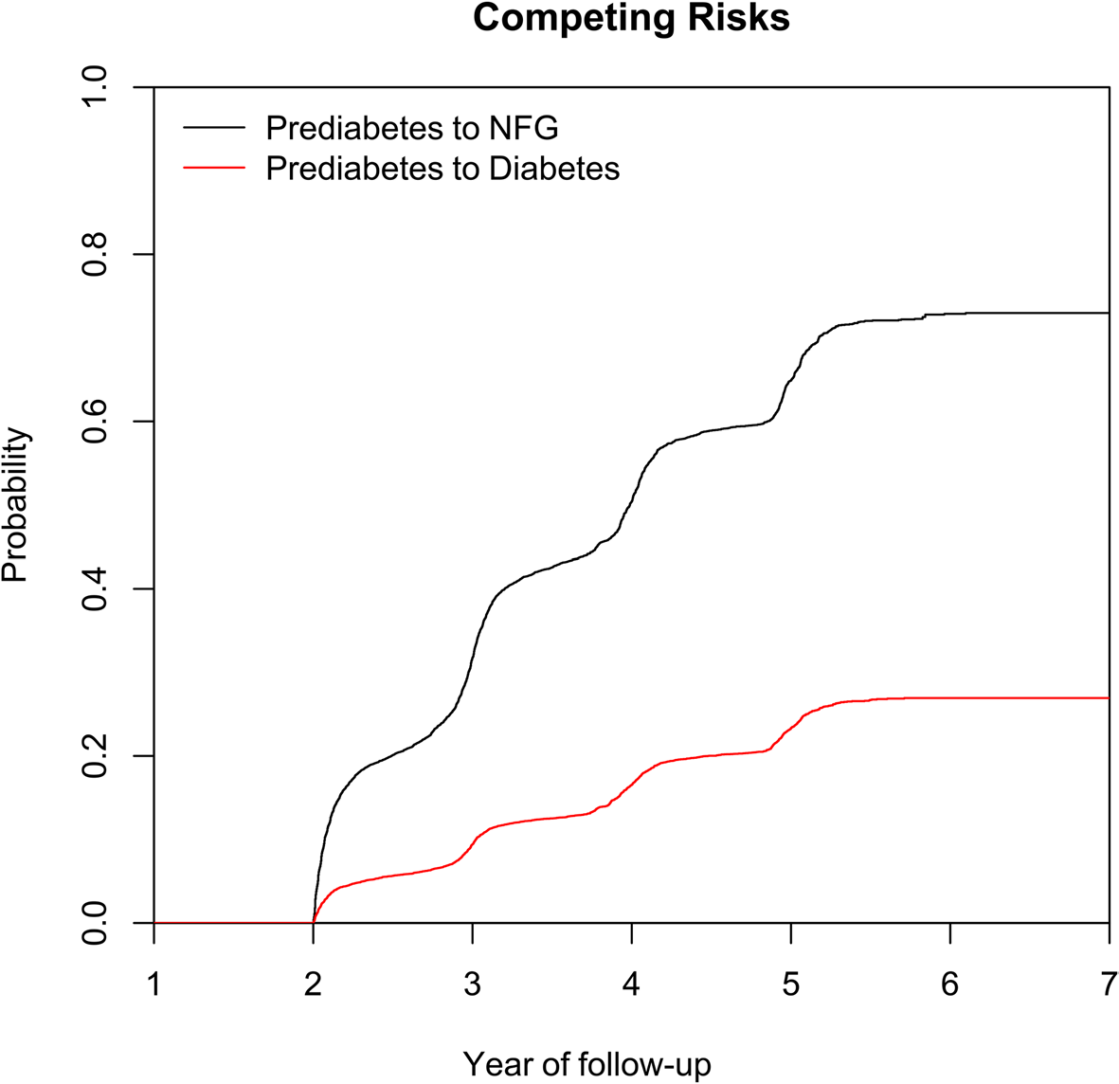
Cumulative incidence curve of prediabetes recovering to NFG or progressing to diabetes

Under the condition where prediabetes progressing to diabetes or recovering to NFG were considered as competing risks, the researchers computed the cumulative incidence rates of prediabetes recovering to NFG or progressing to diabetes using cumulative incidence functions and visualized these competing risks with cumulative incidence curves (Fig. 2). According to the calculations, in the third, fourth, fifth, and sixth years of follow-up, the cumulative incidence rates of prediabetes recovering to NFG were 31.72%, 50.45%, 64.89%, and 72.88%, respectively, and that of prediabetes progressing to diabetes were 9.4%, 16.57%, 23.36%, and 26.93%, respectively. It can be observed that prediabetic participants had a higher likelihood of recovering to NFG compared to progressing to diabetes.

**Fig. 3:**
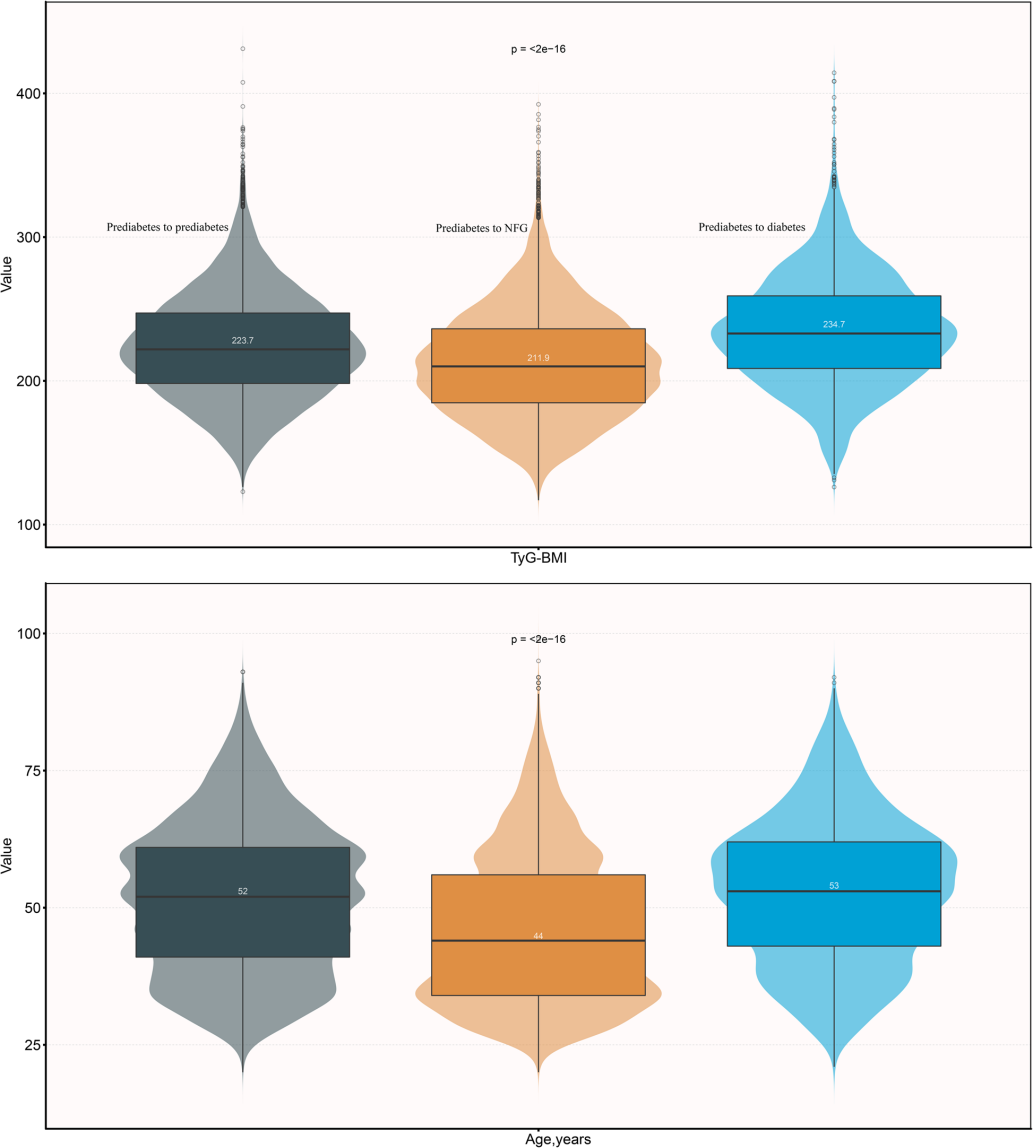
Violin chart showing baseline characteristics of TyG-BMI and age according to glucose status during follow-up

 The baseline characteristics of the study population were summarized at the initial point of inclusion, based on their glycemic status observed during the follow-up period (Table 1). It is noteworthy that, in comparison to participants who remained prediabetic or progressed to diabetes in the future, the researchers observed distinct baseline characteristics among those who later recovered to NFG, as summarized below: (i) Individuals who eventually recovered to NFG had relatively lower baseline values for Cr, LDL-C, BMI, weight, age, TG, BUN, BP measurements, FPG, ALT, TyG index, TyG-BMI, AST, TC, with age and TyG-BMI showing particularly notable differences (Fig. 3). (ii) Among this subgroup, there was a higher proportion of females, a greater prevalence of a family history of diabetes, and fewer individuals with smoking and drinking habits.

**Table 1:** Baseline characteristics summarized according to subjects' glycemic status during follow-up

	**Glucose status during follow-up**		
	Prediabetes	NFG	Diabetes
No. of subjects	10,305	11,278	3814
Sex			
Male	7031 (68.23%)	7087 (62.84%)	2716 (71.21%)
Female	3274 (31.77%)	4191 (37.16%)	1098 (28.79%)
Age, years	52.00 (41.00–61.00)	44.00 (34.00–56.00)	53.00 (43.00–62.00)
Height, cm	166.56 (8.32)	166.75 (8.40)	166.92 (8.28)
Weight, kg	69.40 (61.00–77.50)	67.00 (58.80–75.38)	72.00 (64.00–80.00)
BMI, kg/m^2^	25.08 (3.25)	24.19 (3.31)	25.92 (3.43)
SBP, mmHg	129.12 (17.83)	124.18 (16.77)	131.11 (17.98)
DBP, mmHg	79.46 (11.24)	76.74 (10.77)	80.51 (11.31)
FPG, mmol/L	5.99 (0.32)	5.83 (0.24)	6.15 (0.37)
TC, mmol/L	5.03 (0.95)	4.90 (0.95)	5.07 (0.99)
TG, mmol/L	1.50 (1.02–2.21)	1.28 (0.87–1.92)	1.68 (1.14–2.49)
HDL-C, mmol/L	1.31 (1.13–1.51)	1.34 (1.15–1.54)	1.29 (1.09–1.50)
LDL-C, mmol/L	2.91 (2.47–3.37)	2.84 (2.41–3.33)	2.89 (2.43–3.40)
TyG index	8.89 (0.61)	8.73 (0.62)	9.03 (0.61)
TyG-BMI	223.73 (36.74)	211.89 (37.27)	234.69 (38.41)
ALT, U/L	22.90 (16.00–33.40)	20.30 (14.20–31.00)	25.00 (18.00–38.60)
AST, U/L	24.00 (20.00–29.00)	23.60 (19.60–29.00)	25.00 (21.00–31.70)
BUN, mmol/L	4.91 (4.18–5.80)	4.80 (4.03–5.68)	4.90 (4.13–5.78)
Cr, umol/L	73.10 (62.00–83.72)	72.00 (60.00–82.60)	72.70 (62.00–82.70)
Family history of diabetes	236 (2.29%)	244 (2.16%)	140 (3.67%)
Smoking status			
Current	926 (8.99%)	877 (7.78%)	377 (9.88%)
Past	136 (1.32%)	193 (1.71%)	69 (1.81%)
Never	2362 (22.92%)	2903 (25.74%)	724 (18.98%)
Not recorded	6881 (66.77%)	7305 (64.77%)	2644 (69.32%)
Drinking status			
Current	148 (1.44%)	135 (1.20%)	55 (1.44%)
Past	568 (5.51%)	732 (6.49%)	199 (5.22%)
Never	2708 (26.28%)	3106 (27.54%)	916 (24.02%)
Not recorded	6881 (66.77%)	7305 (64.77%)	2644 (69.32%)

Values were expressed as mean (standard deviation) or medians (quartile interval) or n (%)

*Abbreviations: NFG* normal fasting glucose, *BMI* body mass index, *SBP* systolic blood pressure, *DBP* diastolic blood pressure, *FPG* fasting plasma glucose, *TG* triglyceride, *TC* total cholesterol, *HDL-C*, high-density lipoprotein cholesterol, *LDL-C*, low-density lipoprotein cholesterol, *TyG index* triglyceride-glucose index, *TyG-BMI* triglyceride glucose-body mass index, *ALT* alanine aminotransferase, *AST* aspartate aminotransferase *BUN* blood urea nitrogen *Cr* creatinine

### Evaluating the impact of TyG-BMI on the recovering to NFG or progressing to diabetes in prediabetes

The researchers assessed whether there was multicollinearity between TyG-BMI and other covariates when taking the recovery or progression of prediabetes as the dependent variable. The results were presented in Supplementary Tables 2 and 3. Weight and TC were excluded from subsequent models due to variance inflation factor exceeding 5. Additionally, the researchers generated Schoenfeld residual plots to examine the time-varying effects of TyG-BMI (Supplementary Figs. 1 and 2), and *P*-values for all correlations were higher than 0.05, indicating no violation of the proportional hazards assumption.

Three stepwise adjusted multivariate Cox regression models were used to explore the impact of TyG-BMI on prediabetes recovery/progression (Table 2), it can be observed that in all models, TyG-BMI showed a significant negative association with prediabetes recovering to NFG and a positive association with prediabetes progressing to diabetes [Model III: Prediabetes to NFG (HR): 0.90 (0.88–0.93); Prediabetes to diabetes (HR): 1.26 (1.21–1.31)].

**Table 2:** Multivariate Cox regression analysis of the role of BMI, TyG index, TyG-BMI in assessing changes in glycemic status in patients with prediabetes

	HR (95%CI)				E-value
	Non-adjusted Model	Model I	Model II	Model III	
**Prediabetes to NFG**					
TyG-BMI (per SD increase)	0.80 (0.79, 0.82)	0.84 (0.83, 0.86)	0.86 (0.84, 0.88)	0.90 (0.88, 0.93)	1.46
TyG-BMI (quartiles)					
Q1	Ref	Ref	Ref	Ref	
Q2	0.81 (0.77, 0.85)	0.90 (0.86, 0.95)	0.92 (0.88, 0.97)	0.97 (0.92, 1.02)	
Q3	0.67 (0.64, 0.71)	0.78 (0.74, 0.82)	0.80 (0.76, 0.85)	0.87 (0.82, 0.93)	
Q4	0.59 (0.56, 0.62)	0.66 (0.63, 0.70)	0.70 (0.66, 0.74)	0.82 (0.77, 0.89)	
P-trend	< 0.0001	< 0.0001	< 0.0001	< 0.0001	
**Prediabetes to Diabetes**					
TyG-BMI (per SD increase)	1.43 (1.39, 1.47)	1.40 (1.36, 1.45)	1.38 (1.34, 1.42)	1.26 (1.21, 1.31)	1.83
TyG-BMI (quartiles)					
Q1	Ref	Ref	Ref	Ref	
Q2	1.56 (1.40, 1.74)	1.41 (1.26, 1.57)	1.38 (1.23, 1.54)	1.27 (1.12, 1.43)	
Q3	2.17 (1.95, 2.41)	1.90 (1.71, 2.11)	1.83 (1.64, 2.04)	1.57 (1.39, 1.76)	
Q4	2.81 (2.54, 3.11)	2.52 (2.27, 2.80)	2.39 (2.15, 2.66)	1.82 (1.61, 2.06)	
P-trend	< 0.0001	< 0.0001	< 0.0001	< 0.0001	

Model I adjusted for age, sex and height

Model II adjusted for age, sex, height, family history of diabetes, smoking status, drinking status, SBP, DBP

Model III adjusted for age, sex, height, family history of diabetes, smoking status, drinking status, SBP, DBP, FPG, TG ALT, BUN and Cr

*Abbreviations: HR* hazard ratios, *CI* confidence interval, other abbreviations as in Table 1

**Fig. 4:**
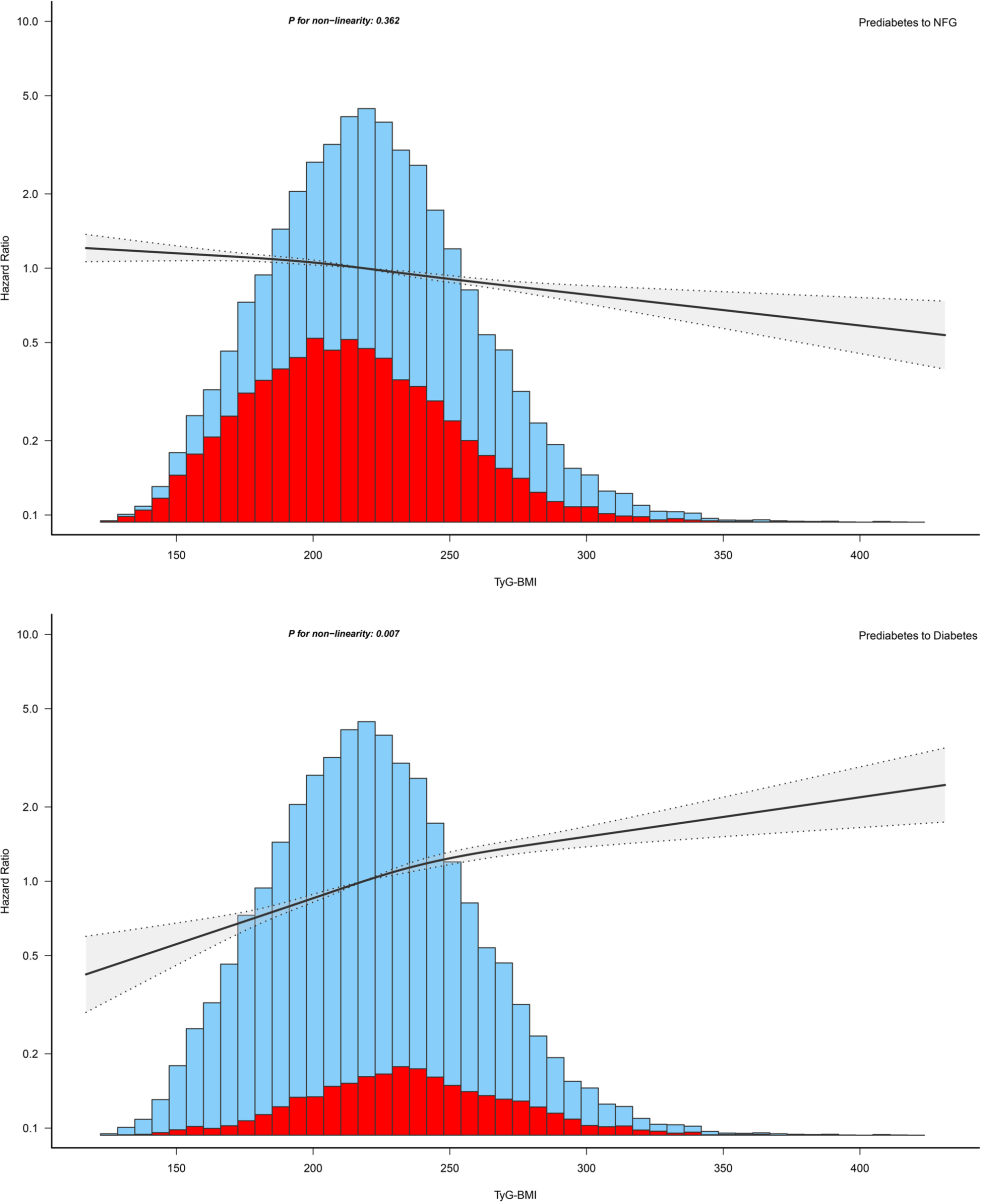
Apply the 4-knots RCS model to fit the dose–response relationship between TyG-BMI and recovery from prediabetes to NFG/progressing to diabetes

The researchers further visualized the dose–response relationship curve of TyG-BMI with prediabetes recovering to NFG/progressing to diabetes based on RCS. As shown in Fig. 4, after adjusting for covariates in Model III, TyG-BMI exhibited a linear negative correlation with prediabetes recovering to NFG (*P*-non-linearity = 0.362) and a nonlinear positive correlation with prediabetes progressing to diabetes (*P*-non-linearity = 0.007).

### Sensitivity analysis

After transforming TyG-BMI as a categorical variable, the new analysis results showed that the negative association of TyG-BMI with prediabetes recovering to NFG gradually strengthened between quartiles (Q1 1 vs Q2 0.97 vs Q3 0.87 vs Q4 0.82), maintaining a negative trend (*P*-trend < 0.0001). In the association between TyG-BMI quartiles and prediabetes progressing to diabetes, the positive association between them gradually increased between quartiles (Q1 1 vs Q2 1.27 vs Q3 1.57 vs Q4 1.82), maintaining a positive trend (*P*-trend < 0.0001) (Table 2). When assessing the study population according to the prediabetes and diabetes diagnostic criteria published by the WHO, it can be observed similar results to the analysis conducted using the ADA criteria (Supplementary Table 4: Sensitivity-1). In the context of considering competing risks, the association of TyG-BMI with the recovery of prediabetes to NFG or progressing to diabetes remained consistent with the main analysis results (Supplementary Table 4: Sensitivity-2). When analyzing people without a family history of diabetes separately, the results were still similar to the ones reported above (Supplementary Table 4: Sensitivity-3). Furthermore, the researchers generated E-values to assess the sensitivity to unmeasured confounding (Table 2), and the results can be considered relatively robust for each SD increment. Finally, 3-knot and 5-knot RCS models were constructed, and the results were consistent with the findings from the 4-knot RCS, indicating a linear negative relationship of TyG-BMI with the recovery of prediabetes to NFG (Supplementary Figs. 3 and 4).

### Subgroup analysis based on common population phenotypes

Regarding different sexes, ages, and BMIs, the researchers conducted subgroup analyses to explore whether there is specificity in the relationship of TyG-BMI with the recovery or progression of prediabetes within these common population phenotypes (Table 3). In these subgroups, it can be observed that BMI and sex did not produce significant interactions with TyG-BMI in relation to prediabetes progression/recovery. However, age showed significant interactions with TyG-BMI in both prediabetes progression and recovery. It's worth noting that in relatively younger populations (< 45 years old), the negative correlation of TyG-BMI with the recovery of prediabetes to NFG weakened, while the positive correlation with the progression of prediabetes to diabetes strengthened. In relatively older populations (≥ 45 years old), the negative correlation of TyG-BMI with the recovery of prediabetes to NFG strengthened, while the positive correlation with the progression of prediabetes to diabetes weakened. In summary, TyG-BMI appeared to be more suitable for evaluating the progression or recovery of prediabetes in younger populations.

**Table 3:** Exploratory subgroup analysis of the role and differences of TyG-BMI in assessing changes in glycemic status in prediabetes patients

	HR per SD de/increase (95%CI)	
	Prediabetes to NFG	Prediabetes to Diabetes
**Sex**		
Male	0.92 (0.89, 0.94)	1.22 (1.17, 1.27)
Female	0.91 (0.87, 0.95)	1.27 (1.19, 1.35)
* P*-interaction	0.7710	0.2870
**Age, years**		
< 45	0.94 (0.91, 0.97)	1.36 (1.28, 1.43)
45–59	0.87 (0.84, 0.91)	1.17 (1.10, 1.24)
≥ 60	0.87 (0.82, 0.92)	1.16 (1.09, 1.23)
* P*-interaction	0.0013	< 0.0001
**BMI, kg/m**^**2**^		
< 24	0.95 (0.90, 1.00)	1.40 (1.24, 1.58)
24–27.9	0.91 (0.86, 0.98)	1.21 (1.10, 1.34)
≥ 28	0.88 (0.81, 0.96)	1.19 (1.08, 1.30)
* P*-interaction	0.2858	0.0813

Models adjusted for the same covariates as in model III (Table 2), except for the stratification variable

*Abbreviations: HR* hazard ratios, *CI* confidence interval, other abbreviations as in Table 1

### Comparing the predictive value of TyG-BMI and its individual components for the recovering to NFG or progressing to diabetes in prediabetes

**Fig. 5:**
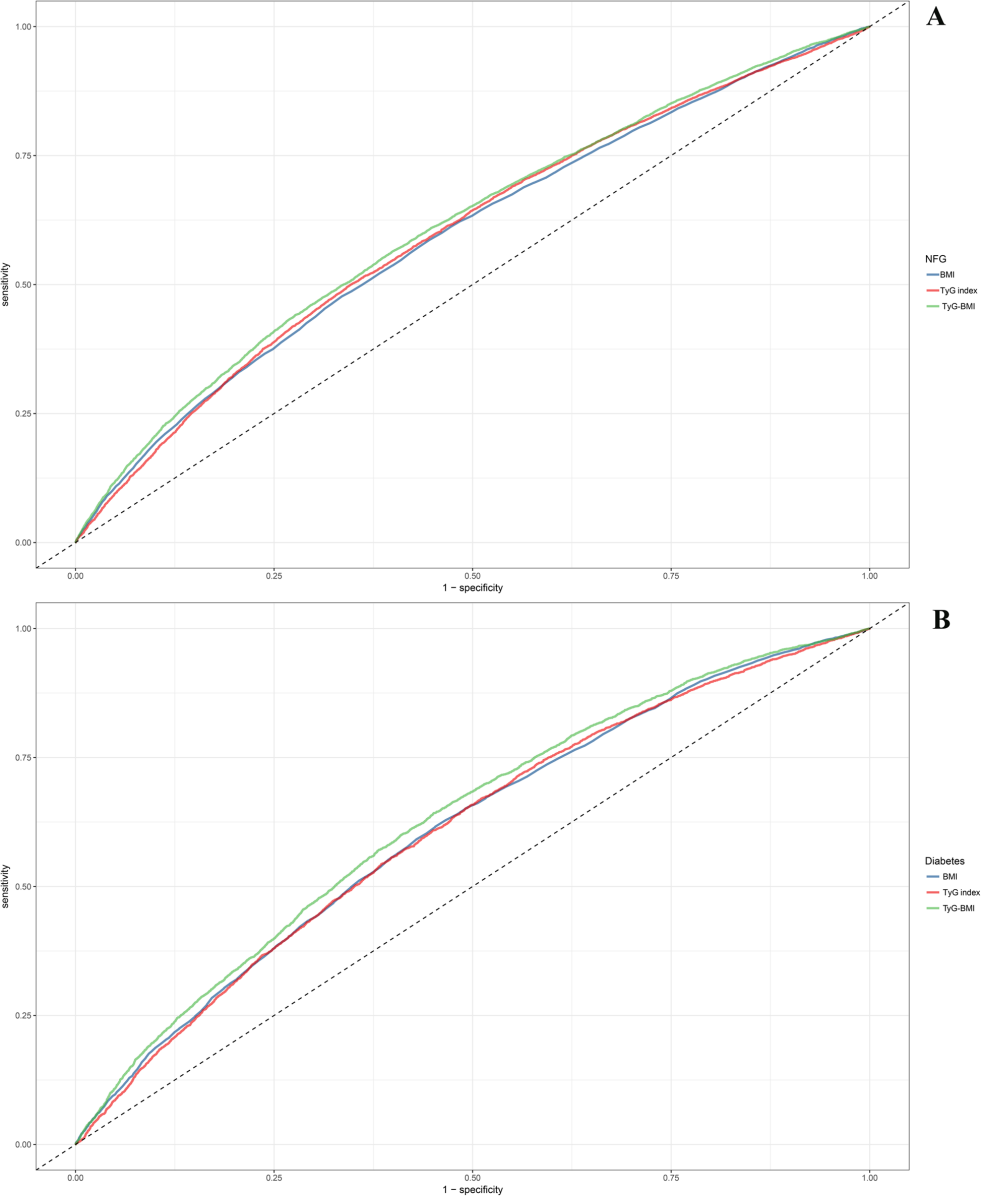
ROC analysis shows the predictive value of TyG-BMI and its components for recovery from prediabetes to NFG/progressing to diabetes

After establishing the impact of TyG-BMI on the recovering to NFG or progressing to diabetes in prediabetes, the researchers conducted further assessments using ROC analysis to assess the predictive value of TyG-BMI and its constituent components. Detailed results from the ROC analysis were presented in Table 4, and they were visualized in Fig. 5. It is evident that TyG-BMI demonstrated a significantly higher predictive value for the recovering to NFG or progressing to diabetes in prediabetes compared to BMI and the TyG index [Prediabetes to NFG (AUC): TyG-BMI 0.61 vs TyG index 0.60 vs BMI 0.59; Prediabetes to diabetes (AUC): TyG-BMI 0.63 vs BMI 0.60 vs TyG index 0.60; all Delong *P* < 0.001]. Additionally, it is worth noting that, in terms of predictive threshold analysis, the thresholds for TyG-BMI and its constituent components for predicting the recovery of prediabetes to NFG (BMI 23.81, TyG index 8.68, TyG-BMI 214.68) were slightly lower than those for predicting progressing to diabetes (BMI 24.82, TyG index 8.79, TyG-BMI 220.27).

**Table 4:** ROC analysis of the predictive value of BMI, TyG index, TyG-BMI on changes in glucose status during follow-up of prediabetes subjects

	AUC	95%CI low	95%CI upp	Best threshold	Specificity	Sensitivity
**Prediabetes to NFG**						
BMI*	0.59	0.59	0.60	23.81	0.66	0.48
TyG index^*^	0.60	0.59	0.61	8.68	0.66	0.50
TyG-BMI	0.61	0.61	0.62	214.68	0.61	0.55
**Prediabetes to Diabetes**						
BMI*	0.60	0.60	0.62	24.82	0.55	0.62
TyG index*	0.60	0.60	0.62	8.79	0.51	0.65
TyG-BMI	0.63	0.62	0.64	220.27	0.55	0.64

*AUC* area under the curve, other abbreviations as in Table 1

^*^P < 0.001, compare with TyG-BMI

## Discussion

This second analysis of a Chinese healthcare cohort indicated that TyG-BMI was negatively correlated with the recovery of prediabetes to NFG and positively correlated with the progression of prediabetes to diabetes. Furthermore, in predicting the recovering to NFG or progressing to diabetes in prediabetes, TyG-BMI exhibited higher impact and predictive value compared to the TyG index and BMI.

Prediabetes, a high-risk state for diabetes, can lead to a range of long-term complications and seriously jeopardize the body's health [1–6, 45, 46]. In previous epidemiological investigations, many researchers have evaluated the role of TyG-BMI in the development of prediabetes, diabetes, and various chronic diseases or adverse outcomes [26–30, 47–54]. In summary, TyG-BMI is a reliable surrogate marker for IR [25], and an elevated TyG-BMI indicates decreased insulin sensitivity, thereby increasing the risk of diabetes and various other chronic diseases or adverse outcomes. In the current study, the researchers further validated the role of TyG-BMI in the development of diabetes, and the results showed that for every one SD increase in TyG-BMI, the diabetes risk increased by 23%. It is important to note that although previous research has made some progress in understanding the role of TyG-BMI in prediabetes and diabetes progression [26–30], it is still unclear how TyG-BMI affects the process of prediabetes recovering to NFG. To address this issue, the current study further analyzed the impact of TyG-BMI on the recovery of prediabetes, and the study results indicated a significant linear negative association between TyG-BMI and prediabetes recovery (*P*-non-linearity = 0.362). Subsequent sensitivity analyses further confirmed the stability of this finding. Additionally, in the current study, the researchers observed a significant interaction with age in the relationship between TyG-BMI and prediabetes recovery/progression. Specifically, this can be explained by a relatively higher risk of TyG-BMI-related prediabetes progressing to diabetes in younger individuals (< 45 years old), and conversely, a younger age is more favorable for prediabetes recovering to NFG in relation to TyG-BMI. The elevated risk of TyG-BMI-related diabetes in younger populations has also been documented in previous studies [26–29]. The underlying reasons for this 'peculiar' outcome may be linked to China's family planning policy, rapid societal development, and accelerated aging. Amidst these burgeoning societal factors, there is a conspicuous decline in the labor force in contemporary Chinese society [55, 56]. Young individuals, who are the primary contributors to the workforce, face substantial societal and psychological pressures compared to the past, which exacerbate their susceptibility to IR [57, 58]. Consequently, this contributes to an increased incidence risk of TyG-BMI-related diabetes. Conversely, the more pronounced advantage observed among young individuals in terms of the recovery of TyG-BMI-related prediabetes to NFG levels is likely primarily attributable to their higher β-cell recovery capacity [59, 60].

Several studies have previously reported on the use of TyG-BMI for predicting prediabetes and diabetes, and, interestingly, the outcomes of these published studies have shown remarkable consistency, indicating that TyG-BMI outperformed both the standalone TyG index and BMI in the prediction of prediabetes and diabetes [26–29]. These congruent findings have been reaffirmed in the current investigation [Prediabetes to diabetes (AUC): TyG-BMI 0.63 vs BMI 0.60 vs TyG index 0.60; All Delong *P* < 0.001]. Furthermore, the researchers conducted an additional assessment to evaluate the predictive value of TyG-BMI and its constituent components for prediabetes recovery; as anticipated, TyG-BMI demonstrated substantial predictive utility in the context of prediabetes recovery, surpassing the predictive performance of the individual TyG index and BMI. These findings further underscored the significant role of TyG-BMI in the prediction of glycemic metabolic disorders. In addition, within the current study, the researchers also assessed the predictive thresholds of TyG-BMI for prediabetes recovery and progression. The results showed that the threshold for predicting recovery of prediabetes was 214.68, while the threshold for predicting progression of prediabetes was 220.27. In contrast, the TyG-BMI prediction threshold for predicting recovery of prediabetes was lower, suggesting that stricter intervention strategies and preventive criteria may be required for prediabetes patients to recover to NFG. To the best of our knowledge, the predictive value of TyG-BMI and its constituent components for the disease trajectory of prediabetes has not been previously reported. In light of the findings from the current study, the researchers believe that TyG-BMI may warrant greater attention in the risk assessment and prediction of glycemic-related disorders.

While research on the recovery of prediabetes is relatively scarce, the findings from the current study, based on cumulative incidence functions, along with the analysis of incidence rates in other ethnic populations [61, 62], suggested that the prediabetic state represents a valuable opportunity, because this phase holds immense potential for recovering to NFG. Moreover, the recovery of prediabetes is expected to significantly reduce the risk of diabetes and various chronic complications [7–17]. Therefore, the benefits of prediabetes recovering to NFG cannot be overstated, and any preventive or intervention strategies that facilitate this unique process may hold substantial clinical significance. The findings from the current study are anticipated to provide clinical practitioners and fellow researchers with valuable insights and assistance in several key areas: (i) In the domain of diabetes prevention, the early evaluation of TyG-BMI among individuals with prediabetes may offer a robust means of identifying the risk of diabetes onset. (ii) For early intervention in prediabetes, monitoring TyG-BMI and maintaining it below 216.68 could potentially expedite the recovery of prediabetes to NFG or increase the probability of recovering to NFG. (iii) In the construction of predictive models for prediabetes recovery, the inclusion of TyG-BMI may substantially enhance the predictive efficiency of these models. (iv) Given that the current study concurrently assessed both the recovery and progression of prediabetes, the results consistently highlighted TyG-BMI as a superior predictive marker compared to the standalone TyG index and BMI. These findings suggested that TyG-BMI could serve as an excellent novel indicator for assessing and screening glycemic-related disorders, and if this can be validated in more studies then it could provide a great boost to reducing the risk of glucose metabolism-related diseases. (v) Although the current study did not explore the mechanisms by which TyG-BMI mediates prediabetes recovery, it is plausible that the resolution of IR plays a pivotal role in this process, which is supported by the fact that TyG-BMI is utilized as a surrogate marker for IR [25], and intervention studies have provided evidence that the improvement of β-cell function is a key factor in prediabetes recovery [22]. Building upon these results and analyses, further assessing the dynamic changes in TyG-BMI in future research will hold significant value in understanding its role in the recovery of prediabetes to NFG, providing robust evidence for clinical validation of mechanisms, and facilitating the development of subsequent preventive strategies.

### Study strengths and limitations

The foremost strength of this study lies in its first revelation of the role and predictive value of TyG-BMI in the recovery of prediabetes to NFG, and the core findings of this research remain robust even under rigorous sensitivity analyses. Furthermore, the large sample size further enhances the reliability of these results.

Several limitations should be duly acknowledged in summarizing as follows: (i) The most prominent limitation may stem from the diagnostic criteria used for study outcomes. As the study population consisted of individuals undergoing routine health screenings at Rich Healthcare Group, only a few participants underwent oral glucose tolerance tests. The present study, under existing circumstances, adopted a method frequently employed in prior research, defining study endpoints solely based on follow-up data of FPG and self-reported diabetes diagnosis [18], potentially leading to an underestimation of the incidence rates of prediabetes and diabetes. Nevertheless, it is noteworthy that the present study consistently yielded concordant results in the main analysis and sensitivity analyses, even at lower incidence rates. Thus, one might argue that the results of the current study are relatively reliable from a reverse perspective. (ii) Given that most participants in the study were of Chinese heritage, caution is advised when extrapolating these results to different ethnic populations. (iii) Following collinearity screening, although AST, HDL-C, and LDL-C all met the model adjustment requirements, their substantial missing values (exceeding 39%) limited the value of imputation and potentially impacted data accuracy [63]. Consequently, to avoid the inability to reflect the authenticity of real-world research due to data missingness, the current research did not include these covariates for adjustment in the multivariable models. As supplementary analysis, this study regards the above-reported variables as unmeasured confounders in the multivariable regression, and further E-value analysis indicated that even in the presence of unmeasured confounding, the results of the current study remained relatively robust. (iv) The current study lacked repeated measurements of TyG-BMI, preventing further exploration of the critical role of IR in the recovery of prediabetes. (v) The follow-up duration in the current study was relatively short, necessitating further research to validate the impact of TyG-BMI on medium- to long-term recovery of prediabetes.

## Conclusion

This large-scale cohort study revealed that the combination of the BMI and TyG index significantly influences the recovery of prediabetic states to normal blood glucose levels, demonstrating substantial predictive value. From the perspective of prediabetes intervention, maintaining TyG-BMI within the range of 214.68 is of paramount significance.

### Supplementary Information

**Supplementary Material 1. **

**Supplementary Material 2. **

## Data Availability

The data set supporting the results of this study has been uploaded to Dryad database (https://doi.org/10.5061/dryad.ft8750v).

## Abbreviations

TyG: Triglyceride-glucose
BMI: Body mass index
NFG: Normal fasting glucose
ADA: American diabetes association
ROC: Receiver operating characteristic
Cr: Creatinine
BUN: Blood urea nitrogen
SD: Standard deviation
VIF: Variance inflation factor
RCS: Restricted cubic splines
AUC: Area under the curve
ALT: Alanine aminotransferase
AST: Aspartate aminotransferase
HDL-C: High-density lipoprotein cholesterol
TC: Total cholesterol
TG: Triglyceride
LDL-C: Low-density lipoprotein cholesterol
FPG: Fasting plasma glucose
BP: Blood pressure
HR: Hazard ratio
CI: Confidence interval
IR: Insulin resistance
