# Supplementary material for: Relative importance of triglyceride glucose index combined with body mass index in predicting recovery from prediabetic state to normal fasting glucose: a cohort analysis based on a Chinese physical examination population
Source: Lipids Health Dis. 2024 Mar 8;23:71. doi: 10.1186/s12944-024-02060-w (PMC10921811; doi:10.1186/s12944-024-02060-w)
Supplement: Supplementary file 2 — Supplementary Material 2. [file 12944_2024_2060_MOESM2_ESM.docx]

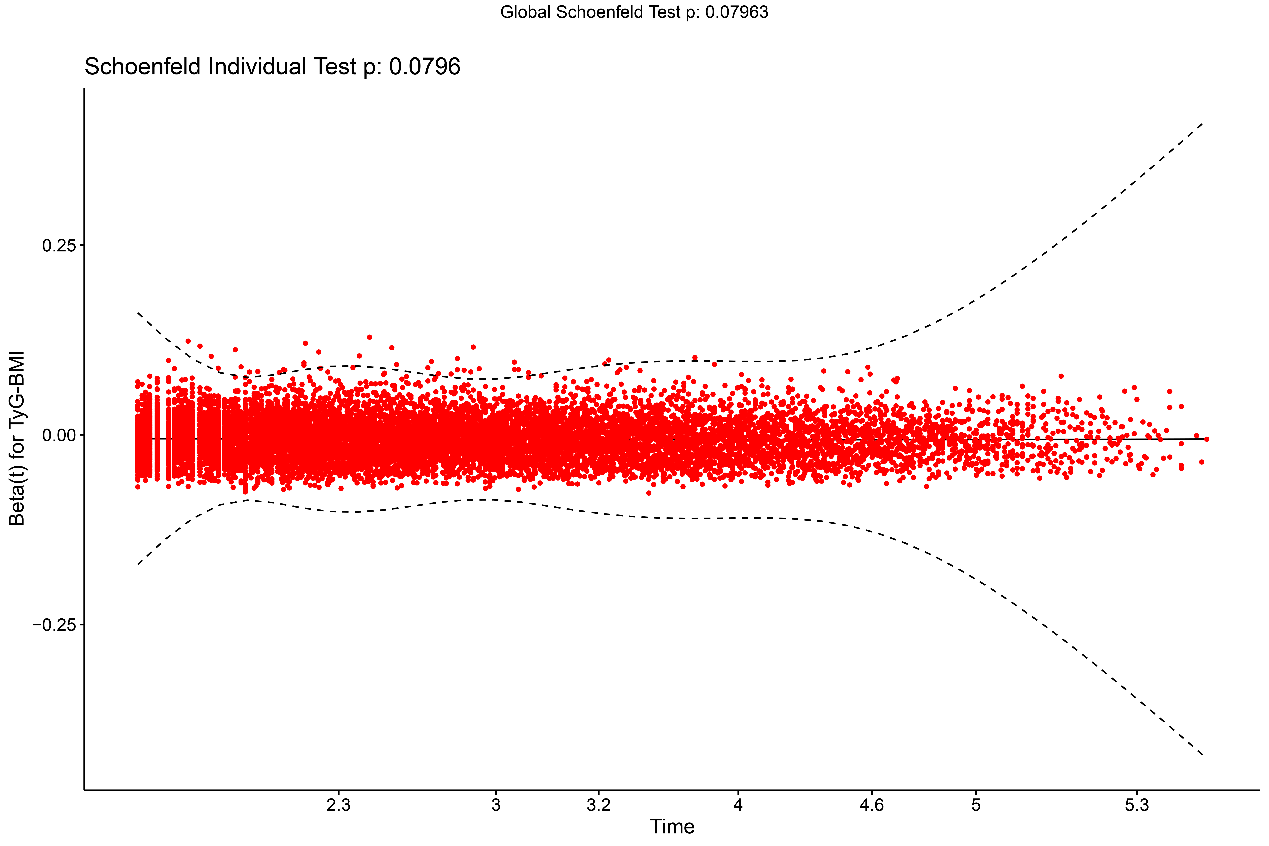


**Supplementary Figure 1**: Schoenfeld residual plot of TyG-BMI changes over time with recovering from prediabetes to NFG as the dependent variable. The p-value of Schoenfeld Residuals Test result is larger than 0.05 which indicated that TyG-BMI is not a time dependent variable and can be analyzed by Cox Proportional Hazards Model.


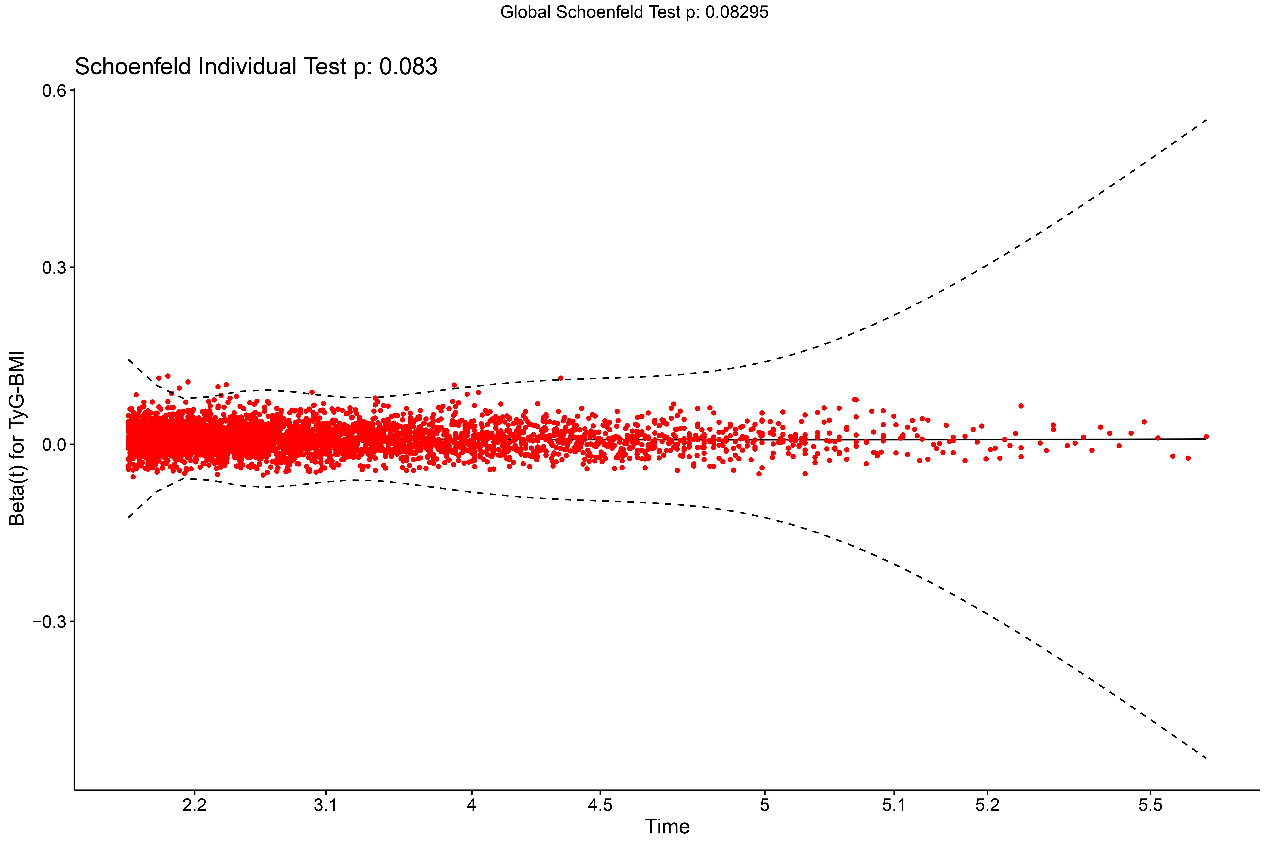


**Supplementary Figure 2:** Schoenfeld residual plot of TyG-BMI changes over time with progression from prediabetes to diabetes as the dependent variable. The p-value of Schoenfeld Residuals Test result is larger than 0.05 which indicated that TyG-BMI is not a time dependent variable and can be analyzed by Cox Proportional Hazards Model.


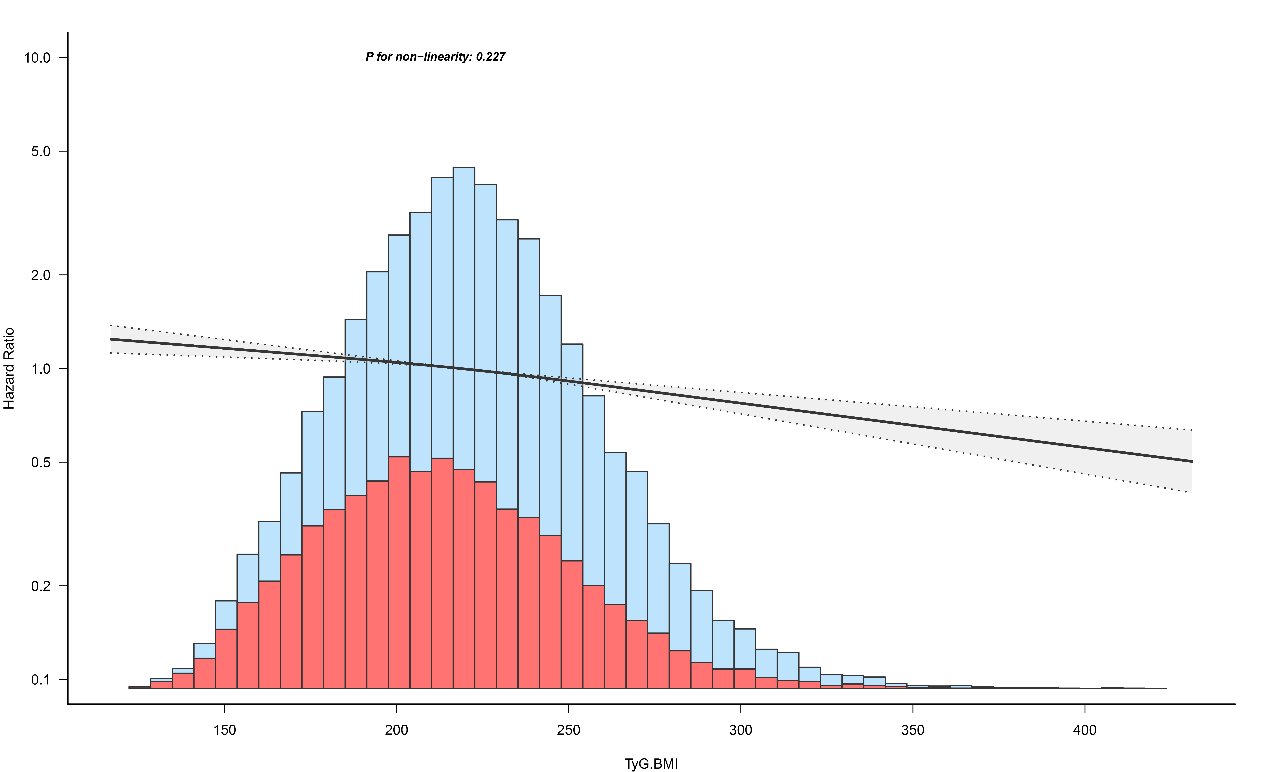


**Supplementary Figure 3:** Apply the 3-knots RCS model to fit the dose-response relationship between TyG-BMI and recovery from prediabetes to NFG.


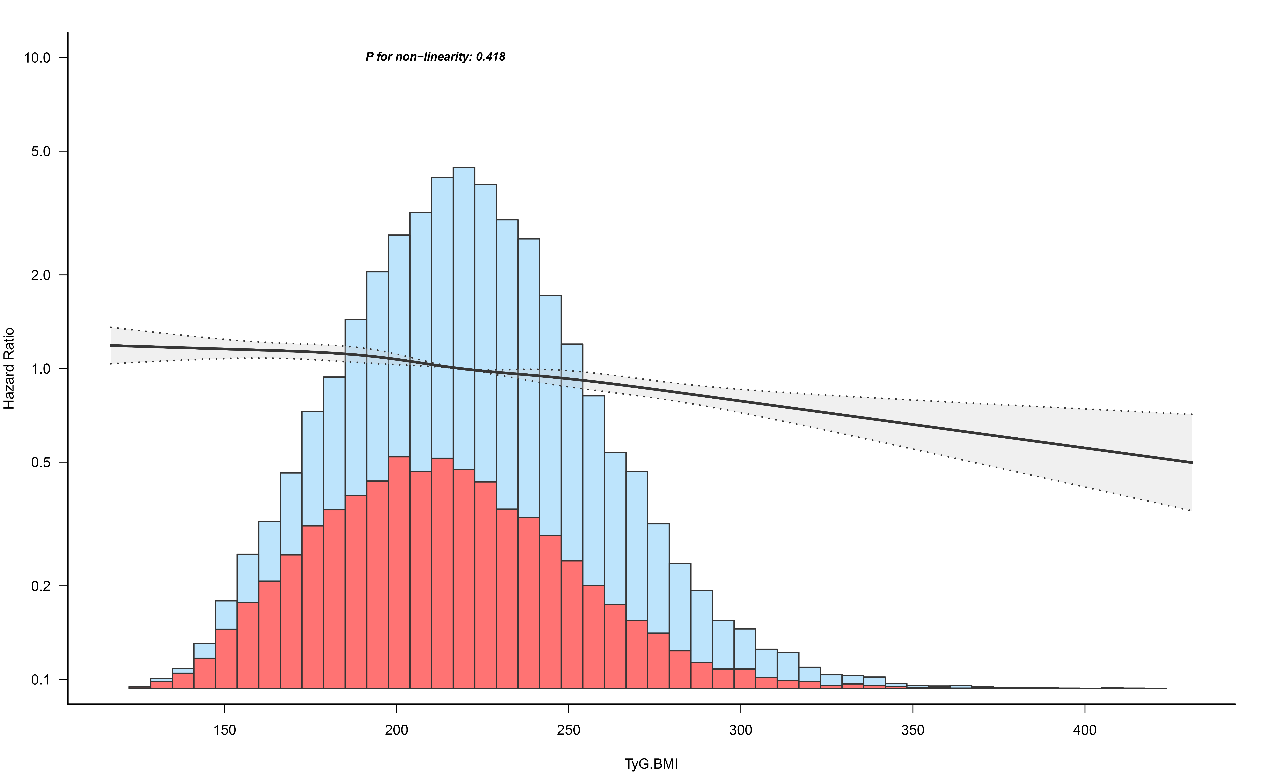


**Supplementary Figure 4:** Apply the 5-knots RCS model to fit the dose-response relationship between TyG-BMI and recovery from prediabetes to NFG.
